# Checkpoint therapy in cancer treatment: progress, challenges, and future directions

**DOI:** 10.1172/JCI184846

**Published:** 2024-09-17

**Authors:** Mesude Bicak, Cansu Cimen Bozkus, Nina Bhardwaj

**Affiliations:** 1 Vaccine and Cell Therapy Laboratory and; 2 Department of Medicine, Division of Hematology and Medical Oncology, Tisch Cancer Institute, Icahn School of Medicine at Mount Sinai, New York, New York, USA.; 3 Parker Institute for Cancer Immunotherapy, San Francisco, California, USA.

In the late 1800s, observations that bacterial infections could lead to tumor regression and that the immune system could recognize and eliminate precancerous cells, later proved by the identification of lymphocytes in the 1950s, shepherded a new era of cancer immunology and immunotherapy. Subsequently, the discoveries of cytokines, IFN-α, and IL-2 and the development of monoclonal antibodies, e.g., B cell–targeting anti-CD20, were pivotal in understanding the regulation of immune responses and established the foundation for targeted immunotherapies. But it is only within the last decade or so that immunotherapy has dramatically affected patient survival rates in many high-incidence cancers, with the development of immune checkpoint blockade (ICB) therapy. Discovery of the immune checkpoint molecules cytotoxic T lymphocyte antigen 4 (CTLA-4), and programmed cell death protein 1 (PD-1) and the development of blocking monoclonal antibodies marked a new era in oncology. The approval of the anti–CTLA-4 antibody ipilimumab for metastatic melanoma in 2011 — a substantial milestone — was followed in 2014 with anti–PD-1 antibodies nivolumab and pembrolizumab to treat advanced or metastatic melanoma and as frontline therapy for high-risk patients in 2016 (Figure 1). ICB has since become a transformative standard of care for multiple types of cancer with currently eight monotherapy and six combination ICB regimens that were FDA approved by 2024 and many that are being tested in ongoing clinical trials.

## Mechanisms of action

Activation and clonal expansion of T cells targeting tumor antigens, including neoantigens, which derive from mutations or alterations unique to tumor cells, is key to initiating an antitumor immune response. T cell activation, mediated by coordinated actions of (i) T cell receptor (TCR) engagement with peptide-MHC molecules on antigen presenting cells (APCs), (ii) costimulation, and (iii) the cytokine milieu, can be inhibited by checkpoint receptor signaling, which helps prevent an excessive immune response that can lead to destructive inflammatory or autoimmune conditions. Although checkpoint receptors are essential for maintaining peripheral tolerance, their expression may be exploited by tumors to ultimately evade immune surveillance. Consequently, blockade of checkpoint signaling represents a powerful strategy for enhancing antitumor immunity. The full extent of how checkpoint receptors exert their inhibitory functions continues to be investigated; however, it is well documented that CTLA-4 blockade primarily acts at the level of T cell priming in the lymph nodes, as it binds to costimulatory receptors on T cells with a greater affinity and avidity than costimulatory ligands CD80 and CD86, impeding the initial activation and proliferation of T cells. PD-1 can also suppress T cell function by inhibiting signaling through CD28 as well as TCR signaling (1). Its blockade relieves the inhibitory signaling, reversing T cell exhaustion and reinvigorating effector function. These distinct mechanisms of actions of CTLA-4 and PD-1/PD-L1 blockade enable their combination, thereby improving therapeutic efficacy, albeit increasing side effects from treatment.

## Mechanisms of resistance

Response rates to ICB therapies vary (between 15% and 100%) depending on the cancer type, patient characteristics, the specific ICB agent used, the unique combination, and the context of administration. The success of ICB is limited by tumor-intrinsic and -extrinsic resistance. Tumor-intrinsic resistance mechanisms include (i) neoantigen depletion and defective antigen presentation; (ii) a low tumor mutational burden (TMB), rendering tumors less recognizable to the immune system, (iii) while a high TMB causes chronic antigen exposure, leading to T cell exhaustion; (iv) genetic alterations in tumor cells, e.g., mutations in JAK or IFNG receptors, disrupting IFN signaling and allowing tumors to evade detection and destruction. Tumor-extrinsic resistance can arise via an immunosuppressive TME with (i) physical barriers, e.g., a dense extracellular matrix or abnormal vasculature causing exclusion of infiltrating immune cells from the tumor; (ii) accumulation of immunosuppressive cell population, e.g., Tregs, myeloid-derived suppressor cells (MDSCs), and tumor-associated macrophages (TAMs); (iii) high levels of immunosuppressive cytokines, such as TGF-β and IL-10; and (iv) metabolic reprogramming of tumors to adapt to nutrient deprivation and hypoxia, which inhibit T cell function. A unique combination of these mechanisms may be relevant for different tumor types causing primary and acquired resistance to ICB.

## Biomarkers

As response rates to ICB vary across tumor types and patients, utilization of biomarkers is key to predicting response, monitoring treatment efficacy, evaluating resistance, and guiding treatment strategies to improve outcomes and minimize side effects. Various biomarkers have been associated with ICB response rates. High levels of PD-L1 expression are associated with a better response in certain cancers. A high TMB is also associated with ICB response due to increased immunogenicity. Accordingly, tumors with mismatch repair deficiency (dMMR), characterized by a high TMB and tumor-infiltrating lymphocytes (TILs), respond to ICB exceptionally well, which led to the accelerated FDA approval in 2017 of pembrolizumab for the treatment of adult and pediatric patients with unresectable or metastatic dMMR solid tumors (2). This case marks the first FDA approval of a drug, based on a common biomarker rather than tumor tissue of origin. Additionally, the presence of TILs, particularly CD8+ T cells and gene expression profiles (GEPs) related to IFN-γ signaling, correlates with better outcomes. A study identified an 18-gene T cell–inflamed GEP that predicts clinical responses to pembrolizumab across multiple tumor types, a signature more robust than PD-L1 expression for predicting clinical benefit (3). These findings highlight the benefit of integrated approaches for biomarker selection while assessing the utility of ICB.Combination therapiesDue to the limitations of ICB monotherapy, combination strategies have been explored for overcoming resistance and improving response rates. Targeted therapies administered with ICB that directly interfere with cancer-promoting pathways, including hormonal therapies, e.g., tamoxifen, have been shown to enhance the efficacy of ICB alone. ICB can also synergize with ionizing radiation (IR) therapy. Preclinical studies revealed that high-dose IR alone could lead to immunosuppression and tumor relapse by inducing PD-L1 expression within the TME. However, the combination of IR with anti–PD-L1 therapy enhanced antitumor immunity by increasing the infiltration and activation of cytotoxic T cells and reducing the accumulation of MDSCs in the tumors, thereby improving tumor control (4). Similarly, ICB combined with chemotherapy also improved survival rates across various cancers, reducing progression risks in small and non–small cell lung cancers (NSCLCs), triple-negative breast cancer (especially PD-L1–positive tumors), head and neck squamous cell carcinoma, esophageal cancer, urothelial cancer, and biliary tract cancer (5).

As multiple checkpoint receptors are often coexpressed in cancer, utilizing dual or triple ICB agents can also overcome resistance. In addition to improved response by ipilimumab and nivolumab combination, dual TIGIT (T cell immunoreceptor with immunoglobulin and ITIM domains) and PD-1 blockade also can improve antitumor CD8+ T cell responses in advanced melanoma patients (6). Other PD-1/PD-L1 inhibitor combinations are also currently being tested in the clinic, including inhibitors for TIGIT (7), LAG-3, and TIM-3. Opdualag, which is a combination of the LAG-3 inhibitor relatlimab with the PD-1 inhibitor nivolumab, is FDA approved for treating unresectable or metastatic melanoma (8).

ICB combined with adoptive cell therapies, such as chimeric antigen receptor (CAR) T cell therapy and TIL therapy, has shown promising clinical efficacy in glioblastoma and enhanced survival rates in malignant pleural diseases, showing potential to elevate remission rates in advanced stages of challenging solid tumors, including melanoma, NSCLC, and ovarian cancer (9). Similarly, neoantigen-based cancer vaccines combined with ICB have improved response rates and overall survival (OS), particularly notable in cancers with high mutational burdens such as melanoma and NSCLC, where neoantigen load correlates with improved outcomes owing to factors such as vaccine-induced priming of neoantigen-specific T cells. This combination results in a broader T cell repertoire and expansion of preexisting T cell populations. Notably, a personalized neoantigen vaccine for high-risk melanoma patients induced strong CD4+ and CD8+ T cell responses, targeting 60% and 16% of neoantigens, respectively. After 25 months, four of six vaccinated patients showed no disease recurrence, and the two with disease progression achieved complete regression with subsequent anti–PD-1 therapy (10). Furthermore, mRNA-based vaccines combined with ICB are leading to substantial tumor reduction and improved progression-free survival (PFS). Developed by BioNTech and Genentech, tested on 16 patients with pancreatic ductal adenocarcinoma, the BNT22 mRNA-based vaccine, combined with atezolizumab and mFOLFIRINOX, induced T cell responses in 50% of patients, leading to longer recurrence-free survival (RFS) (11)​. NEO-PV-01 combined with nivolumab led to robust T cell responses and prolonged disease control in melanoma, NSCLC, and bladder cancer (12). GNOS-PV02 with pembrolizumab also led to tumor regression in metastatic melanoma (12). The combination of neoantigen vaccines with dual checkpoint blockade (i.e., anti–PD-1 and anti–CTLA-4) was further shown to overcome resistance and enhance antitumor T cell activity in early trials, highlighting the potential of combining neoantigen vaccines with ICB therapies (12).

## Adverse events

ICB induces a proinflammatory state of elevated immune activation, potentially leading to immune-related adverse events (irAEs) affecting various organs and tissues. Common irAEs include dermatologic (rash, pruritus), gastrointestinal (colitis), and endocrine (thyroiditis, hypophysitis) disorders. Less frequent but severe irAEs include neurological conditions (peripheral neuropathy, encephalitis), musculoskeletal problems (arthritis, myositis), nephritis, myocarditis, hepatitis, and pneumonitis (13). Management typically involves immunosuppressive treatments (corticosteroids), particularly for severe irAEs, where early intervention is crucial. Notably, corticosteroid use did not affect long-term survival benefits of ICB therapy. irAEs are found to indicate a more robust immune response associated with better treatment outcomes, including improved PFS and OS across various cancer types (14). Studies in NSCLC demonstrated that patients who develop irAEs show higher objective response rates (ORRs), longer PFS, and extended OS (20.5 versus 8.5 months) (15). This pattern also holds true for melanoma, together with higher disease-control rates (15).

## Future implications of ICB

The field of ICB therapy is rapidly advancing, with new therapies and strategies being developed to enhance efficacy, overcome resistance, and minimize adverse events. A crucial aspect of advancing ICB therapy is the need for a deeper understanding of the underlying mechanisms of checkpoint molecules to refine treatment strategies and improve patient selection. Checkpoint inhibitors targeting molecules such as LAG-3, TIM-3, and TIGIT are showing promise in clinical trials. Additionally, next-generation antibodies, including bispecific antibodies and antibody-drug conjugates (ADCs), are being explored to provide more effective antitumor responses (16). Personalized medicine approaches, such as neoantigen vaccines, expanded TILs, and biomarker-driven therapies, aim to optimize treatment outcomes and reduce side effects. Finally, utilizing ICB in the neoadjuvant setting has shown substantial tumor shrinkage, enhanced immune responses, and improved surgical outcomes, promising better long-term survival rates. In a study of 115 patients with nonmetastatic, locally advanced dMMR colon cancer treated with neoadjuvant nivolumab and ipilimumab, 95% achieved major pathological responses, with 68% having a complete pathological response and no disease recurrence over a median follow-up of 26 months, indicating the potential to replace traditional chemotherapy (17). Another study on dostarlimab in 16 patients with dMMR stage II or III rectal cancer showed a 100% clinical response with no disease progression or recurrence over 6 to 25 months, with well-tolerated therapy and no grade 3 or higher adverse events reported (18). Longer follow-up and larger studies are needed to confirm these promising results.

## Figures and Tables

**Figure 1:**
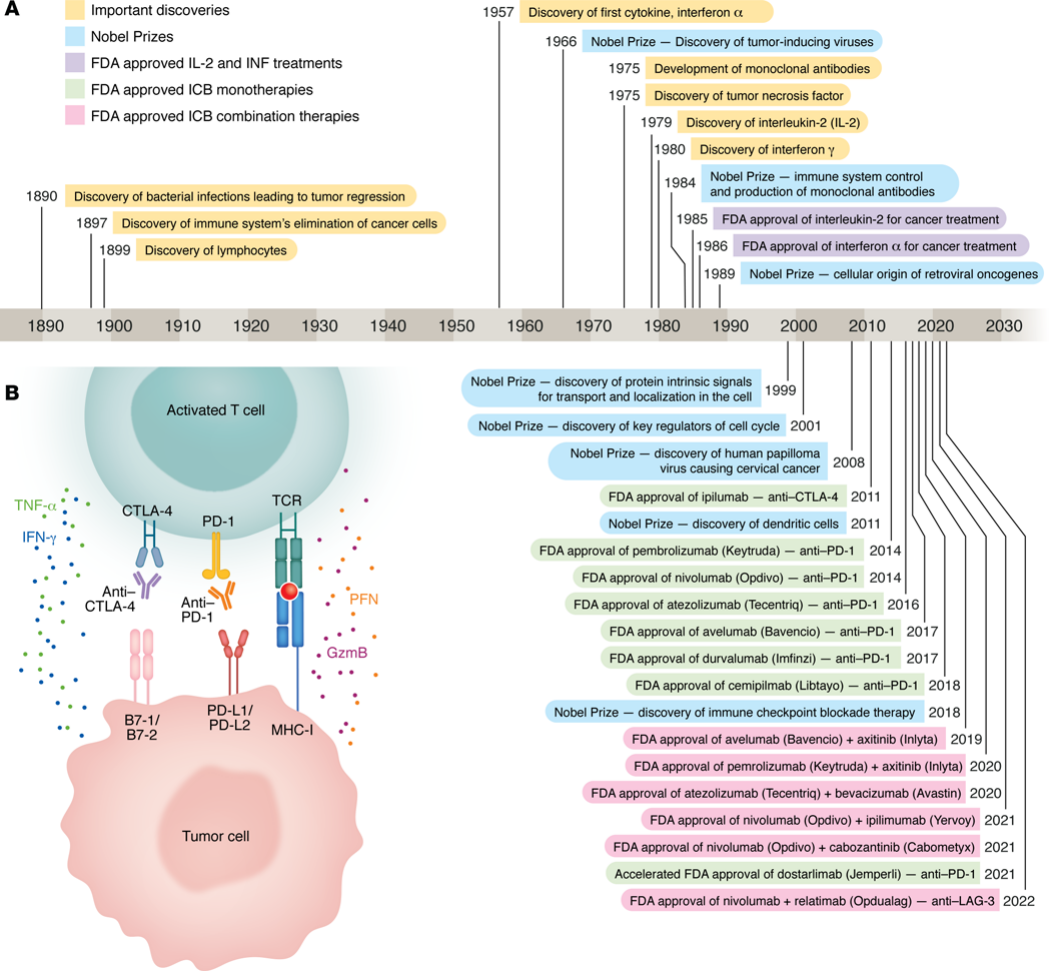
Milestones in cancer research. (**A**) Historical milestones in cancer research include discoveries, Nobel Prizes, and FDA approvals. (**B**) Immune checkpoint blockade with anti–CTLA-4 and anti-PD1 antibodies acts via T cells. Upon binding to their ligands, checkpoint receptors expressed on T cells, including PD-1 and CTLA-4, mediate suppression of T cell–mediated tumor killing. Blocking checkpoint receptor signaling using monoclonal antibodies can restore T cell function, leading to successful tumor killing. PFN, perforin; GzmB, granzyme B.

## Version 1 09/17/2024

Electronic publication
